# An Integrated Monte Carlo Model for Heterogeneous Glioblastoma Treated with Boron Neutron Capture Therapy

**DOI:** 10.3390/cancers15051550

**Published:** 2023-03-01

**Authors:** Leyla Moghaddasi, Eva Bezak

**Affiliations:** 1 Radiation Oncology, Royal North Shore Hospital, Sydney, NSW 2065, Australia; 2 School of Physical Sciences, University of Adelaide, Adelaide, SA 5005, Australia; 3 Cancer Research Institute, University of South Australia, Adelaide, SA 5000, Australia

**Keywords:** BNCT, glioblastoma, CTV, heterogeneous, hypoxic

## Abstract

**Simple Summary:**

Glioblastoma (GBM) is the most aggressive type of astrocytic glioma. GBMs are diffuse infiltrating tumours that present with extensive hypoxia and genetic heterogeneity amongst other features that have rendered treatment strategies ineffectual, despite recent advances in multimodality therapy regimens. The prognosis remains poor and median survival is less than 17 months using adjuvant chemotherapy and X-ray external radiotherapy. Therefore, strategies should be investigated to target complications associated with this malignancy. A targeted approach with high linear energy transfer particles could address infiltration and cellular aggressiveness (e.g., heterogeneity, hypoxia, and intrinsic radiosensitivity) issues, respectively. Boron neutron capture therapy (BNCT), a biochemically-targeted modality, proposes an attractive solution for GBM. A hybrid computational framework was previously developed by our group to quantify the efficacy of BNCT at its current status of development for a simplified GBM model. This work has expanded the framework to a semi-realistic GBM model with heterogeneous radiosensitivity and anisotropic microscopic extensions; moreover, the neutron beam model and neutron transport components have undergone substantial improvements.

**Abstract:**

Background: Glioblastomas (GBMs) are notorious for their aggressive features, e.g., intrinsic radioresistance, extensive heterogeneity, hypoxia, and highly infiltrative behaviours. The prognosis has remained poor despite recent advances in systemic and modern X-ray radiotherapy. Boron neutron capture therapy (BNCT) represents an alternative radiotherapy technique for GBM. Previously, a Geant4 BNCT modelling framework was developed for a simplified model of GBM. Purpose: The current work expands on the previous model by applying a more realistic in silico GBM model with heterogeneous radiosensitivity and anisotropic microscopic extensions (ME). Methods: Each cell within the GBM model was assigned an α/β value associated with different GBM cell lines and a ${}^{10}$B concentration. Dosimetry matrices corresponding to various MEs were calculated and combined to evaluate cell survival fractions (SF) using clinical target volume (CTV) margins of 2.0 & 2.5 cm. SFs for the BNCT simulation were compared with external X-ray radiotherapy (EBRT) SFs. Results: The SFs within the beam region decreased by more than two times compared to EBRT. It was demonstrated that BNCT results in markedly reduced SFs for both CTV margins compared to EBRT. However, the SF reduction as a result of the CTV margin extension using BNCT was significantly lower than using X-ray EBRT for one MEP distribution, while it remained similar for the other two MEP models. Conclusions: Although the efficiency of BNCT in terms of cell kill is superior to EBRT, the extension of the CTV margin by 0.5 cm may not increase the BNCT treatment outcome significantly.

## 1. Introduction

Gliomas are derived from neuroglia or glial cells that—together with neuronal cells—constitute an adult brain and provide critical support to the central nervous system (CNS) [1]. Glioblastoma (GBM), classified as a grade 4 glioma according to the World Health Organisation (WHO) [2], is the most common malignant primary intracranial neoplasm. Due to the aggressive characteristics of GBM, traditional therapies are largely ineffective. The prognosis for GBM patients remains poor, despite major advances in X-ray external beam radiotherapy (EBRT) in the last two decades. According to a recent report, the median overall survival for GBM patients was 9.2 months using concurrent chemoradiotherapy in a cohort of 1479 GBM patients [3]. The median survival showed a mild improvement of 17 months in a population of 233 GBM patients managed with adjuvant radiation therapy and temozolomide [4].

Increasing knowledge of molecular pathways in GBM carcinogenesis and invasion has revealed major characteristic features contributing to difficulties with the management of this malignancy, including intrinsic radioresistance, uncontrolled cellular proliferation, diffusive infiltration, intra- and inter-tumoural heterogeneity, severe intra-tumoural and peritumoural hypoxia, and immune escape. These factors often co-exist or are triggered by one another in the course of malignant evolution. Glucose level and hypoxia (being severe in GBM due to high respiratory activity of brain cells) influence tumour microenvironment (TME) regulation, which in turn, results in the upregulation of enzymes inducing angiogenesis and migration/infiltration [5]. This effect varies between different GBM sub-types (related to different genetic constitutions), which can coexist in the same malignancy [6]. For example, one of the most important diagnostic markers of high-grade gliomas is the status of isocitrate dehydrogenase (IDH) mutation, each associated with different tumour microenvironments and tumour cell metabolism types conveyed by the different gene mutations [7,8]. Glioblastoma with IDH-wild type status has a more aggressive clinical course than the formerly known IDH-mutant GBM [9], now grade 4 astrocytoma [2]. Glioma cells can escape immune response by manipulating the immune functionality of macrophages (summoned to the site of pathological mutations) and recruiting them to create TME, which allows glioma proliferation and invasion [10].

The infiltrative growth pattern and high mobility of GBM cells make the determination of the clinical target volume (CTV) for this tumour particularly difficult. While the uncertainty in its microscopic extension (ME) could result in marginal and distant recurrences, the observed high rate of GBM local relapse [11,12,13,14] can be attributed to its intrinsic radioresistance, hypoxia, and other aggressive features. The dismal prognosis of GBM requires multidisciplinary approaches targeting specific features of this disease for optimal management [15,16].

Boron neutron capture therapy (BNCT) is a targeted type of radiotherapy in which an administered ^10^B agent is taken up preferentially by malignant cells. This technique is based on a nuclear reaction in which a thermal neutron is captured by a stable ^10^B target nucleus, resulting in an emission of a high linear energy transfer (LET) alpha particle (∼1.47–1.77 MeV), a recoiling ^7^Li nucleus (∼0.83–1.01 MeV), and a gamma ray. A list of possible reactions is given in Equation (1). The track length of alpha particles and ^7^Li nuclei range from 4 to 7 μm (LET rate of about 240 keV/μm) [17].

(1) $$\begin{array}{c} \begin{array}{cc} & {}^{1}n{+}^{10}B\to^{11}B^{*}\\ & {}^{11}B^{*}\to^{7}Li{+}^{4}He\;\;\left(6.1\%\;yield\right)\\ & {}^{11}B^{*}\to^{7}Li{+}^{4}He+\gamma \;\;\left(93.9\%\;yield\right) \end{array} \end{array}$$

BNCT has the potential to be effective in the treatment of GBM for three main reasons: (1) better management of intrinsic radioresistance and heterogeneous radio-sensitivities of GBM compared to X-ray EBRT, as the treatment is delivered by high LET radiation, resulting in densely clustered ionisation damages; (2) the process of cell killing in BNCT is less susceptible to oxygen status than EBRT [18]; (3) BNCT, being a biochemical type of radiotherapy, can selectively deliver localised doses to tumour cells while minimising normal tissue toxicity. This is particularly advantageous for GBMs with infiltrative growth patterns and rapid peripheral expansion as it potentially enables the targeting of sub-clinical disease spread into normal brain tissue.

BNCT treatments are complex and limited to a few facilities around the world. The influences of individual physical and biological factors are difficult to measure in practice. In silico simulations are beneficial to assist (even though such models are generally approximations of reality) in de-convolving the contributions of various parameters to clonogenic cell kill and, consequently, the efficacy of BNCT in the treatment of GBM.

**Figure A1:**
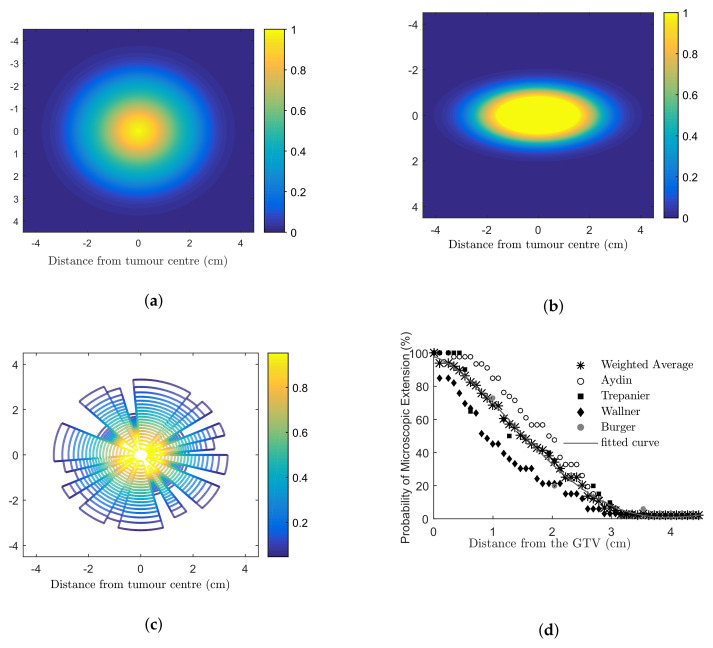
Microscopic extension probability (MEP) distribution models (**a**) isotropic circular; anisotropic (**b**) elliptical; (**c**) irregular models. Dark blue colour (number 0) indicates that the cell is a normal brain cell and the yellow colour (number 1) refers to a cell with 100% probability to being a tumour cell. Colours from blue to yellow (numbers from 0–1) indicate an increasing probability that a cell is a tumour cell. Clinical studies investigating the extent of microscopic extension in GBM patients are shown in (**d**). The PDF fitted to the combined results (a three-term Gaussian function) was considered the basis for the MEP models [19].

In our previous work (see references [19,20]), we developed a simulation platform containing a cellular GBM model, taking into account cellular oxygen status, radiation sensitivity, and microscopic extension distribution called MEP (microscopic extension probability) models (see Figure A1). In the next report from our group, the development and verification of a Monte Carlo (MC) neutron beam model with a realistic energy spectrum for BNCT irradiation was presented [21].

Recently, we reported (see reference [22]) on a BNCT treatment modelling framework for a simplified GBM model consisting of cells with (a) homogeneous radiosensitivity and (b) isotropic distribution of histological infiltration (circular MEP model).

The aim of the current work is to expand the BNCT treatment modelling performed by our group to date by including a more biologically plausible semi-realistic model of GBM that exhibits:

1. Heterogeneous radiosensitivity;
2. Anisotropic infiltration distributions;
3. Comparison of BNCT treatment to the non-uniform oxygen distribution of EBRT;
4. Updated results for homogeneous GBM with isotropic infiltration distribution.

This is to estimate the BNCT treatment efficacy in terms of the number of surviving tumour clonogens for this semi-realistic scenario.

## 2. Materials and Methods

### 2.1. Cell-Based Dosimetry

The geometry architecture designed for the cell-based dosimetry module, described in detail in our previous report [22], was used in this work for a heterogeneous GBM with anisotropic MEPs (see Appendix A). The code was developed using the Geant4 MC toolkit version 4.10.6.p03 (https://geant4.web.cern.ch/, accessed 16 February 2023). The simulated geometry in Geant4 was a 16cm×16cm×10cm brain phantom, encompassing the central section of the GBM model that consisted of two concentric volumes: (1) a spherical gross tumour (1 mm diameter), (2) 4.1 cm ME region from the gross tumour (see Figure A1d) [19], embedded in healthy brain tissue extending 5 cm beyond the field size. The known tortuous paths of neutrons in a biological matter was the rationale for the addition of normal brain tissue to account for scatter radiation contribution, which presents in two areas: (1) in the central region from fast neutrons back-scattered from the deeper layers and thermalised to deposit the dose in shallower depths; (2) penumbra region due to lateral scatter. As this study primarily aims to investigate the effect of the CTV margin extension on the SF, the size of the gross tumour volume (GTV) was intentionally considered small to avoid a substantial CPU time increase. The simulated phantom in the EBRT part of the study replicated that in BNCT.

To simulate the brain injected by a ^10^B agent, the material of each cell in Geant4 was defined as a brain material with an added ^10^B concentration, where ^10^B is a surrogate for the boron agent. The brain material was built in Geant4 using brain elemental composition according to the National Institute of Standards and Technology (NIST) Listings, “compositions of materials used in STAR Databases” web page: http://physics.nist.gov/cgi-bin/Star/compos.pl?matno=123, accessed 16 February 2023. The hydrogen bonding to water molecules in the brain, which significantly affects the slowing down of epithermal neutrons occurring via scattering from hydrogen atoms, was implemented. The distribution of boron concentrations is dependent on whether a cell is a tumour cell or a normal brain cell. Boron concentrations of 13 and 45.5 μg/g (3.5 times larger than normal brain boron concentration) were assumed in normal brain and GBM cells, respectively [22]. Subsequently, using a linear regression, cellular boron concentration was determined as a function of the probability that the cell is a tumour cell (MEP) (i.e., continuous reduction of boron concentration as a function of distance from the GTV):

(2) $$B_{c,ij}=\;32.5\times MEP_{ij}+13$$

where $B_{c,ij}$ is the boron concentration in the ij voxel/cell with $MEP_{ij}$ probability that the cell is a tumour cell. For each MEP model, the corresponding boron bio-distribution was exported to Geant4 to generate the irradiated GBM volume (using the Geant4 nested parametrization method) in which each cell consisted of brain material and the boron concentration as specified by the distribution. Since the exact chemical composition of the agent was not modelled, the conclusions apply to any agent that can deliver a tumour to a normal brain boron concentration of 3.5.

The system was irradiated by our in-house developed BNCT beam model [21], simulating the epithermal spectrum from the LVR-15 reactor at the Rez Research Centre [23]. Nuclear reactor components were not incorporated in the beam model. As a result, the incident neutron beam contained no gamma contamination and the gamma dose was produced from neutron capture by hydrogen atoms only. Two conical beam sizes of 2.5 and 3.0 cm radii were considered to cover PTVs with 2.0 and 2.5 CTV margins, respectively. The model was designed to deliver the treatment in one fraction with a maximum tumour dose of 73.4 RBE-Gy, according to the reported mean maximum tumour doses in the literature [24,25,26,27].

In this work, the current convention in clinical BNCT practices, IAEA-TECDOC-1223 BNCT treatment planning protocol [28], was adopted to translate physical dose to radiobiological dose (RBE-Gy), i.e., each physical dose component (gamma dose, fast neutron dose, thermal neutron dose, and boron dose (sum of lithium and alpha doses)) was weighted by its respective relative radiobiological effectiveness (RBE) factor.

The Geant4 packaged physics list, quark–gluon string with a pre-compound binary cascade high precision neutron (QGSP_BIC_HP) was implemented to simulate particle-tracking by assigning particles with appropriate processes and cross-sections. G4EmStandar dPhysics_option4 was used to simulate photons and electrons and the G4NDL4.2 cross-section library was used for low-energy neutrons. The details on how the employed physics list handles low-energy neutron interactions are described in reference [22]. For this application, we activated the thermal neutron scattering processes for accurate simulations of the thermal neutrons and the neutron fluence rates in the direction transverse to the incident neutron beam. Thermal neutron data libraries were introduced in Geant4 version 4.10.03 and not implemented in the original neutron beam model [21,22], a limitation that was addressed in the current version of the software. In Geant4, a step is considered as a point along the condensed history transport of a particle (the distance between steps may be proportional to the mean free path of the particle). Each step along the transport track stores the information of a particle track at that point (e.g., kinetic energy, total energy deposited, and so forth). In any MC simulation, a step limit should be defined. The size of the step limit has to be sufficiently small to achieve the application’s required precision and should not be too small to increase CPU time unnecessarily. In this application, to ensure that nuclear emissions and fragments (particularly alpha and ^7^Li particles from boron capture reaction with the minimum range of approximately 4 μm) were individually tracked and their corresponding deposited doses were scored, the tracking step size was set to 4 μm. The production threshold/cut-off value for secondary particles was set to 4 μm for $e^{-}$, $e^{+}$, and proton particles, and 0.01 mm for gamma rays. These production thresholds were assigned only to the volume of 9 cm × 9 cm × 0.9 cm size enclosing the scoring slice. Default thresholds of 0.7 mm were used for the rest of the geometry.

Geant4 particle-tracking was performed in the entire geometry and the dose was scored in a single slice with 9 cm × 9 cm× 20 μm dimensions located at 2.0 cm from the phantom surface (approximate depth of dose maximum [21]), perpendicular to the direction of primary beam propagation. In this simulation, to extract cellular absorbed dose from individual particles in the scoring plane, a Geant4 sensitive detector technique was implemented. A parallel geometry was built and linked to the sensitive detector (associated with real geometry) to enable synchronised communication of parallel and real geometries for scoring the absorbed dose in each cell.

Parallel simulations on 8-CPU 64-bit Linux clusters were performed for a total number of $10^{8}$ primary neutrons. Each was run for each MEP model and each beam size (six sets of simulations, three MEP models, two beam sizes). As a result, for each MEP model and beam size, the four dose matrices corresponding to different physical dose components (^7^Li, alpha, gamma, and residual dose consisting of fast neutron and thermal neutron doses) were calculated. The dose matrices for fast and thermal neutron cellular absorbed doses were combined. This was done as the RBE values were the same for these two components.

### 2.2. Survival Fraction Calculation

**Figure 1:**
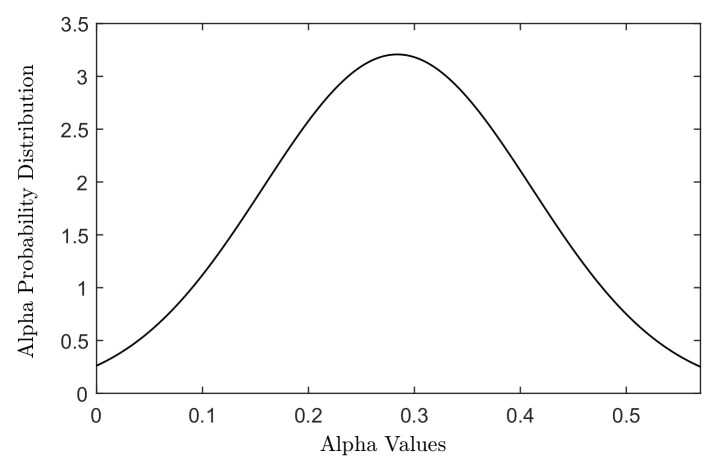
The Gaussian distribution of α values measured for 21 malignant glioma cell lines in the study of Taghian [29].

The cell death was modelled using the linear quadratic (LQ) model, which describes the survival probability of a cell following exposure to a certain dose of radiation. Radiation sensitivity of a cell line is defined in terms of α and β parameters in the LQ model. The distributions of different cellular radiation sensitivities (different α and β parameters) were generated using a Gaussian-weighted distribution of α and β parameters adopted from the in vitro study of Taghian et al. [29] for 21 malignant glioma cell lines, see Figure 1.

For each MEP model, four dose matrices calculated in Geant4 were exported into MATLAB. The total biological dose was calculated and was combined with the MEP models and the radiation sensitivity matrices, representing genetic heterogeneity in terms of radiosensitivity, to obtain the cell survival probability for each cell using Equation (3):

(3) $$SP_{ij}=MEP_{ij}e^{-(\alpha_{ij}d_{ij}+\beta_{ij}d_{ij}^{2})}$$

where $MEP_{ij}$ is the probability that the ij cell is a tumour cell, and $d_{ij}$ is the absorbed dose in the ij cell with radiosensitivity described by $\alpha_{ij}$ and $\beta_{ij}$. $SP_{ij}$ denotes the survival probability of the ij cell. The oxygen enhancement ratio (OER) is assumed to be one for high LET particle radiation [30]. As a result, the OER value of 1 was adopted in this work. Equation (3) reflects the single fraction scheme used for BNCT in this study as opposed to the SP calculation for EBRT with 30 fractions of 2 Gy. To allow a correct comparison, the EBRT scheme reported in our previous work [20] was re-simulated in this work using the extended geometry and updated physics list in Geant4.10.06, hence the corresponding results. Equation (5) was used to calculate the number of surviving cells and SF for the region(s) of interest (ROI):

(4) $$\begin{array}{cc} & \mathrm{surviving}\;\mathrm{tumour}\;\mathrm{cells}\;\mathrm{in}\;\mathrm{the}\;\mathrm{region}=\sum_{i,j}^{}SP_{ij}\left(i,j\in ROI\right) \end{array}$$

(5) $$\begin{array}{cc} & SF_{\mathrm{region}}=\frac{\mathrm{surviving}\;\mathrm{tumour}\;\mathrm{cells}\;\mathrm{in}\;\mathrm{the}\;\mathrm{ROI}}{\mathrm{total}\;\mathrm{number}\;\mathrm{of}\;\mathrm{tumour}\;\mathrm{cells}\;\mathrm{before}\;\mathrm{treatment}} \end{array}$$

**Figure 2:**
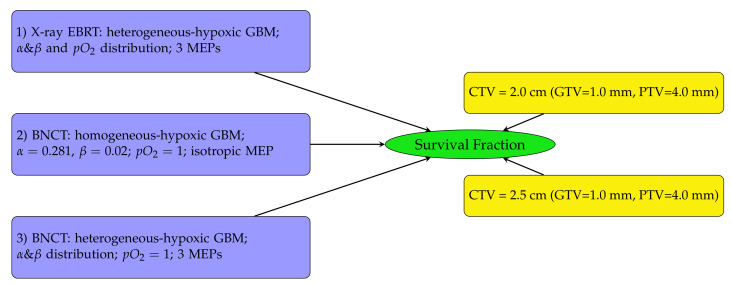
Schematic diagram showing the SF analysis structure. SFs were investigated for three GBM tumour types (left) using two CTV margin extensions (right). In this work, the investigation was conducted for heterogeneous-hypoxic GBM treated with BNCT (number 3 on the left) for both CTV margins on the right and three MEP models. The results were compared with previously reported SFs for heterogeneous-hypoxic GBM using X-ray EBRT [20], and homogeneous-hypoxic GBM using BNCT [22].

The diagram in Figure 2 outlines the SF analysis performed in this work. Heterogeneous-hypoxic GBM treated with BNCT is shown in scenario 3 on the left of Figure 2. The results of this analysis were compared with SFs for heterogeneous-hypoxic GBM using X-ray EBRT [20] (scenario 1), and homogeneous-hypoxic GBM using BNCT [22] (scenario 2). Scenarios 1 and 2 were recalculated in this work. The reason for the re-simulation of scenario 2 is that the thermal neutron data libraries were not implemented in the original neutron beam model, which basically invalidates the previous simulations related to neutron transport/interaction [22]. The enlargement of simulated geometry in this work was the reason for the re-simulation of scenario 1 [20].

Using a 2.0 cm CTV margin, SFs were calculated for each of the three MEP models in several regions (see reference [22] for details): (1) within the beam region (the PTV); (2) within the penumbra region (defined in this study as the region extending 5.0 mm beyond the PTV); and (3) the total SFs (including the in-beam, penumbra, and the out-of-field regions). The results were compared with those calculated for heterogeneous-hypoxic GBM tumour model and all three MEP models, using X-ray therapy published previously [20].

Using a 2.0 cm CTV margin, the differential SFs (defined as the ratio of the number of surviving tumour clonogens to the initial number of tumour cells before treatment in 0.5 mm sphere shells at each distance from the tumour centre) were calculated and plotted for the three MEP models.

To quantify SF reduction as a result of CTV margin extension by 0.5 cm, the Geant4-calculated cell-based dosimetry matrices corresponding to a 6.0 cm diameter beam (corresponding to 2.5 cm CTV margin) were imported to MATLAB and were analysed similarly to obtain SF for each MEP model for the extended CTV margin. The change in SFs ($SF_{change}$), as a result of the CTV increase by 0.5 cm, was quantified using Equation (6):

(6) $$SF_{change}=100\times \frac{SF_{2.0\;cm}-SF_{2.5\;cm}}{SF_{2.0\;cm}}$$

Statistical properties of $SF_{change}$ from multiple runs were evaluated using the MC method. The Matlab script, which was developed in this work, simulated 10 samples within the range of $SF_{change}$ from different runs to derive the expected values and the standard deviations of $SF_{change}$ for different MEP models.

## 3. Results

### 3.1. Assessment of Survival Fractions for a 2.0 cm Clinical Target Volume Margin

Calculated SFs for a 2.0 cm CTV margin are stratified as follows: (a) SF within the beam (the PTV), (b) SF within the penumbra region (5.0 mm beyond the PTV), (c) total SF (including within the beam, penumbra, and out of the beam regions) calculated for circular, elliptical, and irregular MEP models, and for a heterogeneous GBM.

#### 3.1.1. Survival Fractions in Various Regions—2.0 cm CTV Margin

Table 1 summarises the SFs in the regions of interest and the total SFs for the three MEP models. The SF uncertainties are from cellular dose uncertainties from four runs of MC simulations. The SF within the beam was reduced by more than two orders of magnitude for different MEP distributions for BNCT modality as opposed to conventional X-ray therapy. The results indicate a reduction of more than ∼2 times (for elliptical infiltration distribution) in the SF within the penumbra region and the total SF for the simulated BNCT as compared to X-ray EBRT.

#### 3.1.2. Differential Survival Fraction—2.0 cm CTV Margin

**Figure 3:**
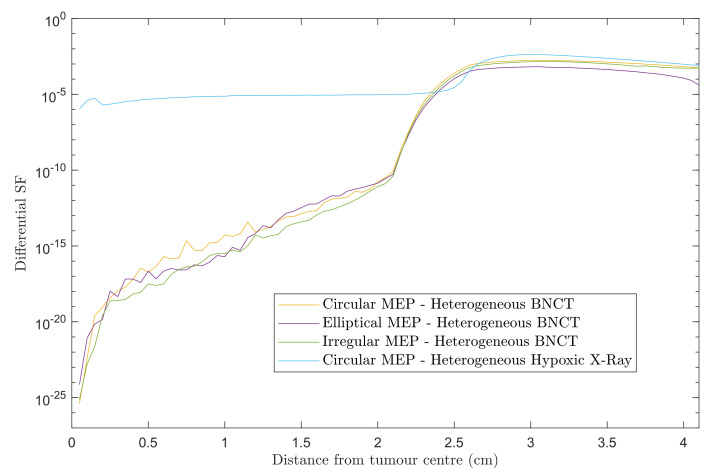
Differential SFs versus distance from the tumour centre for two scenarios: heterogeneous-hypoxic GBM models treated with (1) BNCT applying circular, elliptical, and irregular MEP distributions; and (2) conventional X-ray therapy for a circular MEP model. The graphs were obtained using 2.0 cm CTV margin (corresponding to 2.5 cm beam radius).

Figure 3 shows differential SF curves in 0.5 mm steps using heterogeneous GBM models for the three MEP distributions treated with BNCT. For comparison, the results for heterogeneous-hypoxic GBM with circular MEP distribution treated with conventional X-ray therapy is presented. Note that the results presented here are based on the re-simulation in this work and differs slightly in the penumbra region from our previous report [20]. A marked decrease in the SF within the beam was observed for BNCT as compared to X-ray EBRT, irrespective of the pattern of microscopic extension distribution. For all three MEP models, the in-beam SF for BNCT gradually increased with distance from the tumour centre prior to reaching the penumbra region, while the SF remained almost constant in the entire extent of beam area for the GBM model treated with X-rays. This can be attributed to the dose gradient within the irradiated volume for BNCT treatment due to the negative gradient of boron concentration (see reference [22]), and the boron dose component as a consequence. For the heterogeneous GBM model, the in-beam SFs show similar trends with a variation in values between different MEP models, and are considerably larger than those for the homogeneous GBM [22].

### 3.2. Quantification of Survival Fraction Reduction following Clinical Target Volume Margin Extension

**Figure 4:**
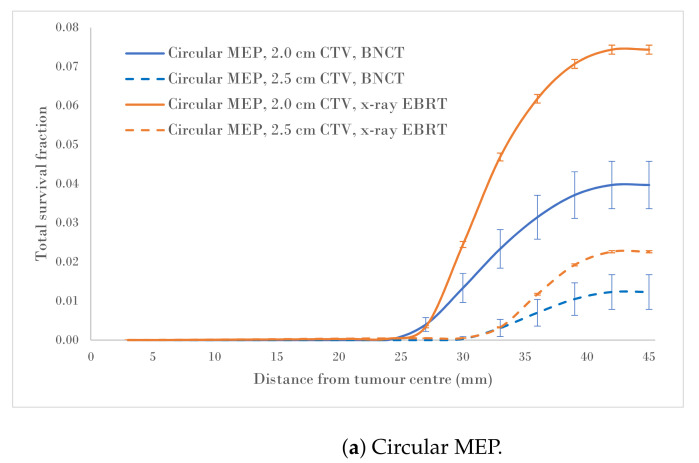

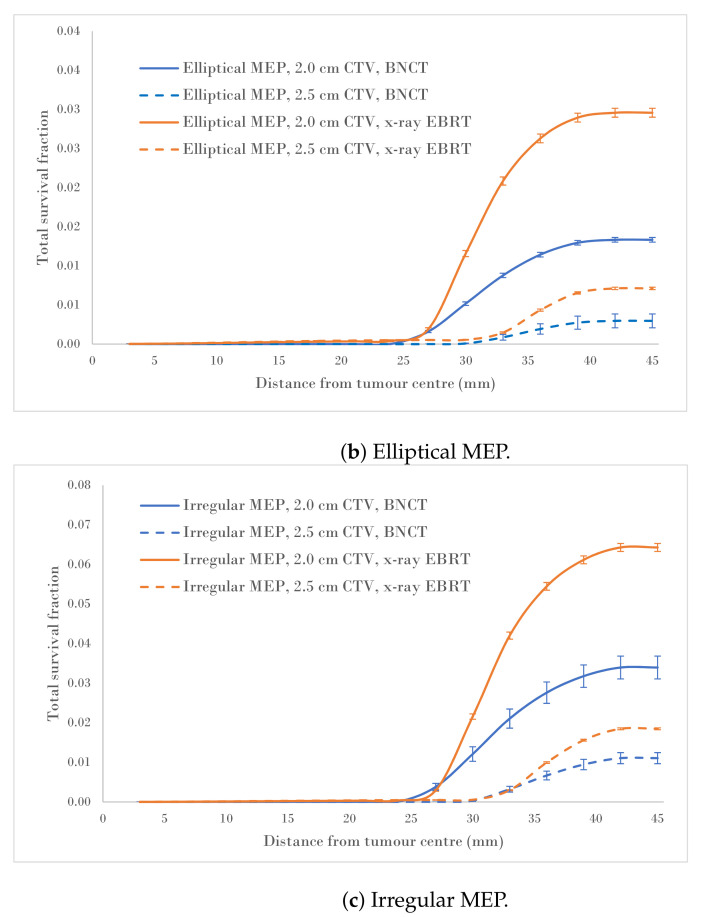
Total SF versus distance from the tumour centre for 2.0 and 2.5 cm CTVs applying three microscopic extension distribution models for a heterogeneous-hypoxic GBM treated by BNCT as compared to conventional X-ray therapy. The SF uncertainties (±1σ) are from cellular dose uncertainties from four runs of MC simulations.

Figure 4a–c show a comparison of the total survival fractions corresponding to a 2.0 cm CTV margin (5.0 cm PTV) and a 2.5 cm CTV margin (6.0 cm PTV) for the circular, elliptical and irregular MEP models and for the heterogeneous-hypoxic GBM model, respectively.

The calculated $SF_{change}$ data, as a result of the CTV margin increase by 0.5 cm, are summarised in Table 2. As suggested by these results, the SFs for heterogeneous-hypoxic GBM treated with BNCT and 2.5 cm CTV margin were reduced between (62.9±3.9)% and (76.9±3.0)% for irregular and Elliptical MEPs, respectively, compared to the 2.0 cm CTV margin. The reduction in the SF as a result of the CTV extension for heterogeneous-hypoxic GBM treated by conventional X-ray therapy ranged from (69.7±0.3)% to (76.1±0.6)% for circular and elliptical MEP models, respectively. The improved SF as a result of the CTV margin extension was significantly lower for BNCT as compared to conventional X-ray EBRT for irregular MEP (*p*-value = 0.0001), while the difference between the two modalities remained insignificant for the other MEP distributions.

## 4. Discussion

The model developed in this work is an expansion on the BNCT simulation of the simplified GBM model, with a homogeneous cellular population and isotropic infiltration, described in our previous report [22]. In the current work, genetic heterogeneity in terms of radiosensitivity was incorporated into the cellular platform of the GBM model and three different infiltration distributions (circular, elliptical, and irregular MEPs) were investigated. The model aimed to evaluate the cell kill efficacy for the simulated BNCT and the results were compared with those for X-ray EBRT. Additionally, the effect of the CTV margin extension on cell survival was investigated.

The calculated SFs of the simulated BNCT of heterogeneous hypoxic GBM, the calculated SFs within the beam region varied slightly between the MEP models (Table 1). Although the overall SF values within the beam were found similar to those for conventional X-ray EBRT, the pattern of survival was completely different between these two modalities (Figure 3). Differential SF curves presented in Figure 3 allowed for the analysis of the SF within the beam area and beyond in more detail. For the simulated BNCT, SF gradually increased as a function of distance from the tumour centre as a result of a reduction in boron concentration. The differential SF trended similarly for all MEP models. For the simulated X-ray EBRT, however, the SF trend changed completely, showing a plateau within the beam with a local peak within the GTV, which received an additional boost of 10 Gy in X-ray EBRT (30 fractions of 2 Gy to the PTV plus a 10 Gy boost to the GTV) [20]. This suggests that the tumour most likely relapses in the GTV when treated by conventional X-ray EBRT, while BNCT should be able to provide local tumour control in dense clonogenic regions (i.e., the GTV). Therefore, in terms of tumour control within the PTV, the gain from BNCT is expected to increase as the size of the GTV is increased. Compared with a heterogeneous GBM, homogeneous GBM showed a larger SF gradient within the beam, suggesting a superior response of a GBM with the homogeneous cell population to BNCT, which unfortunately is not realistic in most cases. The incorporation of heterogeneity and anisotropic microscopic extension did not alter the pattern of SF considerably but still suggests reduced efficacy of BNCT as compared to homogeneous scenarios. It is difficult to directly compare these simulation results with clinical findings due to the fact that the data addressing post-treatment failure analysis for BNCT is limited in the literature. However, the differences between calculated SF patterns in this work explain the varying failure patterns for BNCT and X-ray therapy reflected in published literature. For example, as reported by Aydin [31] and Lee [32], for the simulated X-ray EBRT, the GBM tumour relapsed locally and particularly in the GTV region with severe hypoxia (consistent with the local peak in Figure 3). The SF gradient within the beam for the simulated BNCT indicates that the tumour is likely to recur at farther distances from the GTV, which is in agreement with the observation in the study of Matsuda et al. [33]. Although the SF in the penumbra is lower for all three MEP models using BNCT as compared to X-ray EBRT, it is still significant and could increase the risk of marginal recurrence, as also demonstrated in published clinical studies [24,34]. Published clinical data, while being scarce for BNCT failure patterns, are in agreement with this simulation finding. In a cohort of 8 GBM patients treated by BNCT with the median follow-up duration of 20.3 months in the study of Matsuda et al. [33], none of the patients recurred within the GTV and 6 out of 8 patients recurred in the low dose regions of CTV (where the average minimum dose dropped to about half of that in the GTV region) and the failure was attributed to low or heterogeneous boron concentration and insufficient thermal neutron delivery to the CTV. Similarly, the local tumour progression occurred only in three cases in a cohort of 20 high-grade meningioma patients with the median follow-up duration of 13 months [24].

The SFs in the penumbra region were also evaluated in this work to quantify the contribution of histological disease to total SFs for various microscopic extension distributions. The ratio of the SF in the penumbra region to total SF, which is an approximate measure of the ME contribution to SF, for the simulated BNCT, ranged from ∼0.5 to 1.5% between MEP models, while it varied from 1.1 to 2.4% for MEP models using X-ray EBRT. The large variation between MEP models in terms of the SF in the penumbra region during BNCT treatment could be attributed to neutron scatter. Fast neutrons that have scattered laterally into the penumbra region and thermalised through multiple scattering, could undergo a boron neutron capture reaction, which depends on the boron concentration in the tumour periphery and therefore the MEP model. As summarised in Table 1, the SF in the penumbra and the total SF for BNCT were significantly lower compared to corresponding SFs for the simulated X-ray EBRT. Additionally, the simulation results suggest that BNCT may be the most efficacious for tumours with elliptical (elongated) microscopic extension.

Analysis of SF reduction vs. CTV margin extension (performed in the current work) could be useful for clinicians when balancing local control with normal tissue toxicities. As shown in Figure 4a–c, BNCT results in markedly reduced SFs for both CTV extensions for all three MEP models as compared to conventional X-ray therapy for heterogeneous-hypoxic GBM. As shown, the response of GBM to BNCT is the best for elliptical infiltration distribution (Figure 4b) and the least favourable for irregular microscopic extension (Figure 4c). It was observed that BNCT does not offer an advantage over X-ray EBRT in terms of the reduction in SF as a result of the CTV margin extension and is significantly less for irregular MEP, see Table 2). This indicates that while the extension of the CTV margin could be beneficial and prolong the survival of GBM patients treated by X-ray EBRT by reducing the risk of marginal recurrence, it may not be of significant benefit to GBM patients treated with BNCT. That is, BNCT, at its current development stage, fails to offer an improved prognosis for GBM as the infiltrative growth cannot be targeted without toxicity.

The efficacy of BNCT depends on the selective uptake of a boron agent by tumour cells or, in other words, on the ratio of tumour to normal tissue boron concentration as well as the number of thermal neutrons arriving at the tumour site. Introduction of accelerator-based BNCT (AB-BNCT) technology has shown promise to overcome the latter by producing superior epithermal beam quality compared to reactors [35,36,37] and cyclotron-based AB-BNCT is already clinical in Japan [38,39]. Various types of AB-BNCT, including electrostatic radio frequency quadrupole (RFQ), have been proposed and are at different stages of progress in Argentina [40], Japan [41,42], Russia [43], Finland [44], Israel [45], and others.

Therefore, the synthesis of a suitable boron agent that can selectively deliver sufficiently high concentration of ${}^{10}B$ to the entire population of tumour cells and sustain for the duration of the irradiation while depleting reasonably fast from normal tissue to reduce toxicity is the outstanding roadblock to circumvent. The quest for new boron delivery agents with optimal bio-distribution and toxicology for clinical use has remained the subject of considerable research, resulting in the synthesis of hundreds of new BNCT agents [36,46]. Thus far, none of the new-generation agents have demonstrated convincing selectivity and toxicology profiles to warrant human trials. Solutions to the above-highlighted radio-pharmaceutical challenges could lay the foundation for future clinical trials and possible clinical translation. For more information on novel BNCT agents, please refer to reference [46].

The model developed in this work is a semi-realistic and flexible hybrid model, integrating the MC dose calculation with an analytical cell survival algorithm to study the radiation effects in individual cells rather than the average over the entire tumour. The platform established in this work can be used for further GBM investigations or it can be modified to other tumour sites. A useful application will be to conduct a sensitivity study on the impact of different tumours on normal tissue boron concentrations on the SF. The results of such a study can guide the determination of the optimal tumour to normal brain boron concentration ratio.

This model still has limitations, which should be addressed in future research. Although particles were tracked across the entire geometry, due to computational limitations, the cellular absorbed dose was only scored in a 2D plane at the depth of dose maximum (2.0 cm). That is, the resulting SF for the simulated BNCT is an underestimation of real SF with a 3D GBM tumour. In addition, the calculated absorbed dose in the penumbra region is systematically lower than expected due to a reduction in the thickness of the simulated phantom (hence, an incomplete scatter contribution) and that the beam production line is not incorporated into the beam model. This results in an overestimation of SF in the penumbra region in this study, which counteracts the effect of the aforementioned limitation.

The use of a modified LQ model to convert physical dose to biological endpoint (cell death) is another limitation of this model. Radiation-induced cell death is a complex phenomenon involving a large number of factors, such as immunogenesis, the tumour microenvironment, repopulation, and many more that are not taken into account in the LQ model. Furthermore, the validity of the LQ model, which is derived based on the assumption that hits/lesions are Poisson-distributed, is theoretically questionable at large doses, as is the case for BNCT. As a result, the cell survival for the simulated BNCT is underestimated by the LQ model [47,48]. A potential extension of this model is to replace the LQ model used in the software package developed in this work with a more advanced mathematical cell death model incorporating molecular level parameters including repair probabilities of simple and complex DNA double-strand breaks (DSBs), e.g., lethal and potentially lethal (LPL) [49] or two-lesion kinetics (TLK) [50] models. This requires the further development of the MC component to simulate track structure and DNA damage. Thereafter, the clustering algorithm designed by Douglass et al. [51] can be used to obtain DSBs. Nevertheless, at present, the application of these models to this project is not possible as the available MC software (capable to simulate particle track structure) does not support boron material and neutron interactions. Therefore, the development of more accurate cell survival models is subject to further developments of Geant4-DNA [52] or RITRACKS [53].

## 5. Conclusions

In this work, we investigated the responses of in silico heterogeneous GBM of various infiltration geometries to BNCT. The results in terms of surviving tumour cells were compared with those obtained previously for X-ray EBRT. Unlike X-ray therapy, which cannot overcome radioresistance and complexities of GBM, BNCT could potentially be a promising modality for this malignancy under these conditions: (1) the ability of a boron agent to selectively deliver sufficiently high concentrations of ${}^{10}B$ atoms to the entire clonogenic population; and (2) the availability of a suitable neutron beam with a high proportion of thermal neutrons while minimising fast neutron components.

The main outcomes of this study are:

1. The holy grail of radiotherapy is to maximise the tumour control while the toxicity to the surrounding normal tissue is kept at a minimum. It is evident that increasing the CTV margin improves tumour control, however, it is useful to estimate the gain when two competing goals are sought after. A quantification tool was developed to estimate the reduction in survival fraction due to the extension of the CTV for three MEP distributions. It was concluded that the reduction in SF, which was lower than that for X-ray therapy for all MEP models, may not justify the increased normal brain exposure to high LET radiation.
2. It was demonstrated that BNCT is efficacious for GBM within the beam in terms of cell kill efficacy. Any increase in the dose and the CTV margin extension should be subject to further improvements in the boron micro-distribution and the neutron beam spectrum, and should be evaluated with care.

## Figures and Tables

**Table 1:** Survival fractions (SFs) in different regions for heterogeneous-hypoxic GBM model, using three MEP distributions, for BNCT and conventional X-ray EBRT.

Modality	BNCT		
MEP	SF within the Beam (%)	SF within the Penumbra Region (%)	Total SF (%)
Circular	0.04±0.05	1.29±0.32	3.97±0.68
Elliptical	0.02±0.002	0.50±0.01	1.33±0.03
Irregular	0.03±0.02	0.99±0.16	3.13±0.28
	**Conventional X-ray EBRT**		
Circular	0.042±0.01	2.41±0.07	7.43±0.16
Elliptical	0.043±0.01	1.11±0.03	2.96±0.08
Irregular	0.042±0.01	2.11±0.06	6.41±0.13

**Table 2:** Changes in the SFs (Equation (6)) as a result of the CTV extension (from 2.0 to 2.5 cm) for a heterogeneous-hypoxic GBM model and the three MEP models treated with BNCT and conventional X-ray EBRT.

MEP Model	Circular	Elliptical	Irregular
	**BNCT**		
SF with 2.0 cm CTV (%)	3.97±0.68	1.33±0.03	3.13±0.28
SF with 2.5 cm CTV (%)	1.23±0.44	0.26±0.09	1.11±0.16
$SF_{change}\left(\%\right)$	70.5±2.3	76.9±3.0	62.9±3.9
	**Conventional X-ray Therapy**		
SF with 2.0 cm CTV (%)	7.43±0.16	2.97±0.08	6.43±0.13
SF with 2.5 cm CTV (%)	2.27±0.03	0.71±0.02	1.86±0.02
$SF_{change}\left(\%\right)$	69.7±0.3	76.1±0.6	71.1±0.5
*p*-value	0.29	0.41	0.0001

**Figure A2:**
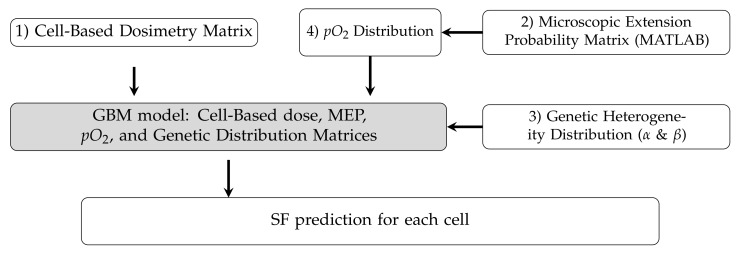
Schematic diagram showing the structure of the model components designed in the previous work [20].

## Data Availability

All data needed to replicate our analyses are available upon request from the corresponding author.

## Abbreviations

The following abbreviations are used in this manuscript:

AB-BNCT	accelerator-based BNCT
BNCT	boron neutron capture therapy
CNS	central nervous system
CTV	clinical target volume
DSB	double-strand break
EBRT	External beam radiotherapy
GBM	glioblastoma
GTV	gross tumour volume
LET	linear energy transfer
LQ	linear quadratic
MEP	microscopic extension probability
MC	Monte Carlo
OER	oxygen enhancement ratio
PTV	planning target volume
RBE	radiobiological dose
SF	survival fraction
TME	tumour microenvironment
TLK	two-lesion kinetics

## Appendix A. An Overview of the Progression of GBM Treatment Modelling

The GBM treatment modelling framework of the current work was developed for a GBM composed of a population of cells with individual radiosensitivities and oxygenation levels (including hypoxia) treated with 6 MV X-ray EBRT [20]. The GBM model was designed as a 2D matrix comprised of both tumour and normal brain cells, with each being assigned a tumour clonogenic probability, oxygen status, radiosensitivity, and the calculated absorbed dose.

The cell-based dosimetry component was developed using Geant4 (version 4.9.6.p04) to calculate the absorbed dose in each cell within a spatial GBM model. The dimensions of the geometry were designed such that it fully encompassed the extension of the GBM microscopic disease in all directions. To enable dosimetry at the cellular level, the volume was divided into 20 μm cubic voxels.

The microscopic extension probability (MEP) models used in this work were developed in MATLAB ® (Mathworks, Natick, MA) to simulate the infiltration of tumour cells into the healthy brain using a probability density function. A probability distribution function (PDF) was obtained based on published clinical data addressing recurrence patterns of GBM (Figure A1d). This PDF assigned each cell with a probability of being a tumour clonogenic as a function of distance from the gross tumour. Based on the PDF, three 2D distributions of MEP were developed: (1) circular MEP where microscopic infiltration occurred isotropically in all directions according to the clinical function (Figure A1a); (2) elliptical MEP, which simulated the preferential diffusion of GBM histological disease along the white matter fibre tracts [54] (Figure A1b); and (3) irregular MEP developed by using two random number generators to determine the radial infiltration extension and a polar angle (Figure A1c).

The individual matrices (calculated absorbed dose, MEP, radiosensitivity, and hypoxia) can be combined to predict cell survival probabilities, and subsequently, cell survival fractions. The details were described in reference [19]. Figure A2 schematically shows the individual components of the integrated model. The details are described in reference [20].
